# Supplementary material for: Extracting the Information Backbone in Online System
Source: PLoS One. 2013 May 14;8(5):e62624. doi: 10.1371/journal.pone.0062624 (PMC3653959; doi:10.1371/journal.pone.0062624)
Supplement: Appendix S1 — Appendix to the manuscript. Figure S1, The variation tendencies of , and with the macro-step increases. step- is named the identifier of th macro-step. The results of Netflix are shown in sub-figures (Netflix-1), (Netflix-2) and (Netflix-3), and those of Movielens are shown in sub-figures (Movielens-1), (Movielens-2) and (Movielens-3). This figure focuses on the time-aware algorithms. Figure S2, The variation tendencies of , and with the macro-step increases. step- is named the identifier of th macro-step. The results of Netflix are shown in sub-figures (Netflix-1), (Netflix-2) and (Netflix-3), and those of Movielens are shown in sub-figures (Movielens-1), (Movielens-2) and (Movielens-3). This figure focuses on the topology-aware algorithms. (PDF) [file pone.0062624.s001.pdf]

Supporting Information

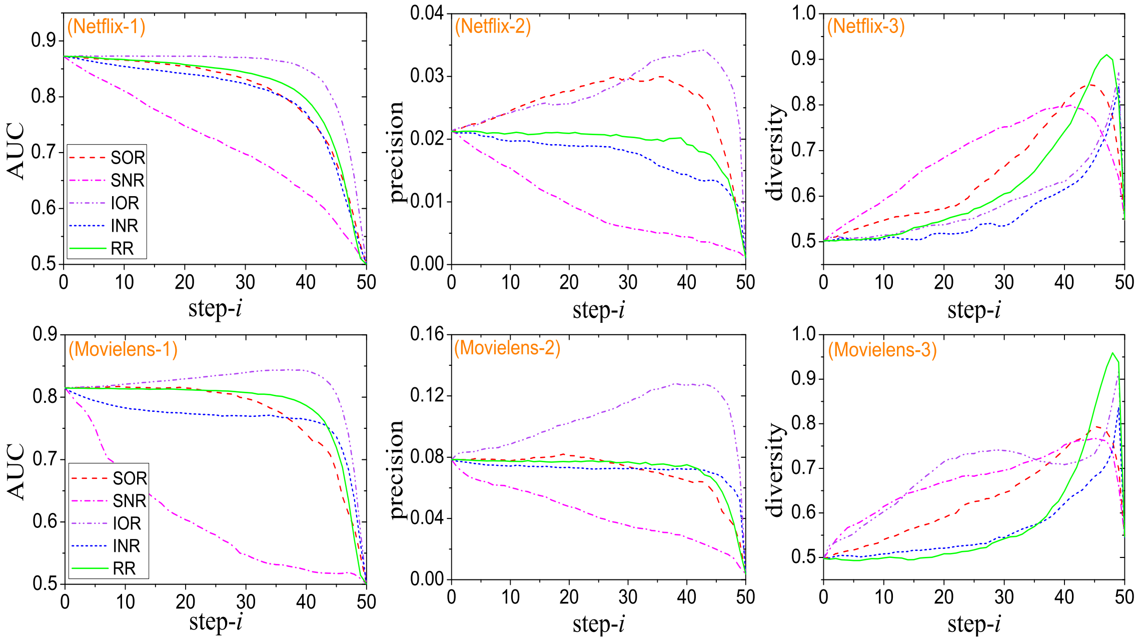

Figure S1. The variation tendencies of AUC, $P(L)$ and $H(L)$ with the macro-step increases. step-i is named the identifier of ith macro-step. The results of Netflix are shown in sub-figures (Netflix-1), (Netflix-2) and (Netflix-3), and those of Movielens are shown in sub-figures (Movielens-1), (Movielens-2) and (Movielens-3). This figure focuses on the time-aware algorithms.

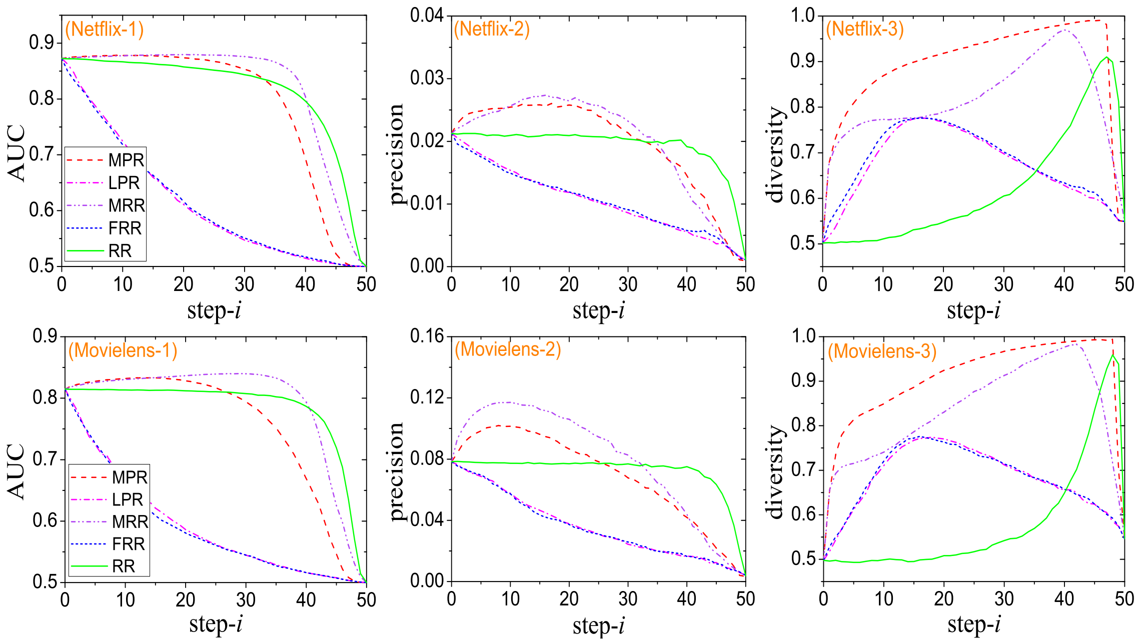

Figure S2. The variation tendencies of AUC, $P(L)$ and $H(L)$ with the macro-step increases. step-i is named the identifier of ith macro-step. The results of Netflix are shown in sub-figures (Netflix-1), (Netflix-2) and (Netflix-3), and those of Movielens are shown in sub-figures (Movielens-1), (Movielens-2) and (Movielens-3). This figure focuses on the topology-aware algorithms.
